# Polyunsaturated Fatty Acids Drive Lipid Peroxidation during Ferroptosis

**DOI:** 10.3390/cells12050804

**Published:** 2023-03-04

**Authors:** Michael S. Mortensen, Jimena Ruiz, Jennifer L. Watts

**Affiliations:** School of Molecular Biosciences, Washington State University, Pullman, WA 99164, USA

**Keywords:** ferroptosis, lipid peroxidation, *Caenorhabditis elegans*, PUFA, MUFA, DGLA, AA

## Abstract

Ferroptosis is a form of regulated cell death that is intricately linked to cellular metabolism. In the forefront of research on ferroptosis, the peroxidation of polyunsaturated fatty acids has emerged as a key driver of oxidative damage to cellular membranes leading to cell death. Here, we review the involvement of polyunsaturated fatty acids (PUFAs), monounsaturated fatty acids (MUFAs), lipid remodeling enzymes and lipid peroxidation in ferroptosis, highlighting studies revealing how using the multicellular model organism *Caenorhabditis elegans* contributes to the understanding of the roles of specific lipids and lipid mediators in ferroptosis.

## 1. Introduction

Ferroptosis as an iron-dependent non-apoptotic form of regulated cell death that occurs when cellular antioxidant systems are overwhelmed [1]. Many excellent reviews have chronicled the hallmarks of ferroptosis: redox active iron, polyunsaturated fatty acid (PUFA)-containing phospholipids and lipid peroxide repair enzymes [2,3,4,5]. Ferroptosis has generated wide interest because it is associated with a variety of illnesses and diseases, including neurodegenerative diseases such as Alzheimer’s disease [6,7] and Huntington’s disease [8], several types of cardiovascular disease (reviewed in [9]), diabetes and diabetic complications [10,11,12] and ischemia-reperfusion injuries of the kidney and liver [13,14,15]. As such, an important goal in the study of ferroptosis is the development of inhibitors to combat diseases such as these. On the other hand, several ferroptotic mechanisms overlap with tumor suppressor pathways, such as modulation by the p53 RAS/MAPK pathway [16,17,18] and radiation-induced tumor suppression [19]. Thus, the induction of ferroptosis could be harnessed to suppress tumors. A key to the development of treatments for diseases involving ferroptosis is the elucidation of the specific lipids that act to trigger and propagate ferroptotic cell death.

## 2. Lipid Peroxides Are Hallmarks of Ferroptosis

Early work in ferroptosis focused on key roles of the phospholipid glutathione peroxidase 4 enzyme (GPX4) [20]. The GPX enzymes use the reduced form of glutathione (GSH) as a cofactor to convert lipid hydroperoxides on phospholipids into lipid alcohols, which limits the levels of peroxidation in a cell [21,22]. Their major protective function in preventing ferroptosis in both in vivo and in vitro models has been well documented [20,23,24,25,26]. Some of the first ferroptosis small molecule inducers act as inhibitors of the GPX system. For example, Erastin inhibits the uptake of reduced glutathione into the cell, thereby lowering the activity of the GPX4 enzyme, while RSL3 was shown to directly limit the functionality of GPX4 [23]. The key role of GPX enzymes in the protection against ferroptosis pointed to oxidized phospholipids as drivers of ferroptotic cell damage [24].

Lipid peroxides are extremely damaging to cells because they disrupt the thickness, permeability and structure of membrane bilayers [27,28]. Using modeling and experimental data, a current hypothesis is that during ferroptosis, membrane thinning and increased curvature drive increased accessibility to oxidants and pore formation, resulting in membrane destruction [29]. Ferroptotic damage is characterized by a cell swelling effect that is propagated through cell populations in a lipid peroxide- and iron-dependent manner [30]. In addition to membrane fragility, lipid peroxide breakdown products, including 4-hydroxynonenal (HNE) and malondialdehyde (MDA), are damaging to cellular processes, because they form adducts with proteins and DNA [31,32]. PUFAs are common components of phospholipids that are highly susceptible to lipid peroxidation because of their multiple double bonds arranged such that hydrogens can be extracted from the acyl chain more readily than from saturated or monounsaturated fatty acyl groups [33]. Lipid peroxidation can occur enzymatically, by enzymes such as lipoxygenase (LOX), cyclooxygenase (COX) and cytochrome P450s (CYPs), or it can occur non-enzymatically, by free radical-induced peroxidation, autoxidation and photodegradation [34]. 

## 3. Non-Enzymatic Lipid Peroxidation 

**Figure 1:**
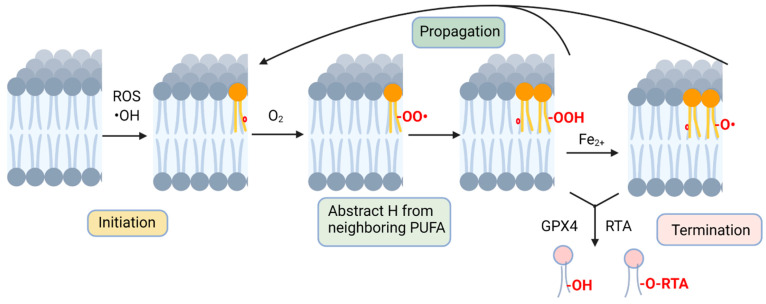
Non-enzymatic lipid peroxidation. Peroxidation is initiated by cellular ROS, where hydroxyl, alkoxyl or peroxyl radicals abstract a hydrogen from a PUFA acyl group (radical electrons denoted as red circle). A PUFA peroxide is formed by reacting with molecular oxygen and abstraction of a hydrogen from an adjacent membrane PUFA. Fenton chemistry contributes to further lipid radical formation, contributing to the chain reaction of lipid radicals attacking acyl groups on nearby unsaturated phospholipid molecules. Lipid peroxidation is terminated by actions of radical-trapping antioxidants or by reduction by catalyzed by glutathione peroxidase activity. Figure created with BioRender.com, accessed on 1 February 2023.

Non-enzymatic lipid peroxidation occurs in three distinct stages [35] (Figure 1). The first step, initiation, is the formation of a radical molecule from a membrane PUFA that is triggered by interactions with radicals, such as hydroxyl radicals (•OH), leading to the abstraction of a hydrogen from the carbon chain [36]. This leaves a free electron in the fatty acid, which causes the rearrangement of electrons, provided by a nearby double bond, to a more favorable position. The molecule that results from this process is known as a lipid radical [36], which reacts readily with molecular oxygen to form a lipid peroxyl radical. In the second step of the lipid peroxidation mechanism, propagation, this newly formed lipid peroxyl radical extracts a hydrogen from a neighboring PUFA molecule, and the H is added to the lipid peroxyl radical, generating both a lipid hydroperoxide and a new lipid radical. The new radical mirrors the first steps of the initiation reaction, with the PUFA losing a hydrogen, resulting in electron rearrangement and the formation of a lipid radical and a lipid peroxyl radical on the neighboring PUFA [37]. This creates a chain reaction of lipid peroxidation within a membrane. The third and final step of this process, known as termination, occurs when lipid radicals and peroxides are resolved, either by reduction with glutathione peroxidases or by the action of radical-trapping antioxidants [38,39].

## 4. Radical-Trapping Antioxidants (RTA) Are Potent Ferroptosis Inhibitors

A potent category of small molecule ferroptosis inhibitors are radical-trapping antioxidants (RTAs), which prevent the propagation of lipid peroxidation [38]. The ferroptosis-specific inhibitor ferrostatin-1 (Fer-1) works through its N-cyclohexyl moiety to link itself to the lipid membrane to scavenge nearby lipid ROS [1,40,41]. Liproxstatin-1 (Lip-1) is a spiroquinoxalinamine derivative that acts as an RTA by lowering the levels of lipid peroxidation in the lipid membrane [40,41]. Lip-1 was first identified as a specific ferroptosis inhibitor in a molecular screen, and was shown to lower signs of oxidation in both human cells and a mouse model [26]. Vitamin E, a collection of antioxidants such as alpha-tocopherol [42] and its water soluble form, Trolox, work as RTAs to lower ferroptosis in vitro [1,43] and in vivo [26]. Bazedoxifenem, a drug known as a modulator for estrogen receptors [44], was identified as an inhibitor of ferroptosis. Bazedoxifene suppresses ROS formation in erastin2-treated human cells by acting as an RTA [45].

## 5. Iron Accumulation Leads to Ferroptosis in Aging *Caenorhabditis elegans*

Cellular iron contributes to the initiation and propagation steps of non-enzymatic membrane lipid peroxidation. Cellular iron participates in the Fenton reaction that generates highly reactive hydroxyl and peroxyl radicals from cellular hydrogen peroxide. These radicals then abstract hydrogens from membrane PUFAs to form lipid hydroxyl radicals that participate in the propagation of lipid peroxidation until they are terminated. Two important factors of ferroptosis are the opposing axis of action between the peroxide-reducing behavior of glutathione and the ROS-generating influence of iron. The necessity of this balance is illustrated in *Caenorhabditis elegans*, wherein an age-dependent increase in iron is correlated with a decrease in glutathione, leading to ferroptosis of intestinal cells in aging worms. Inhibiting this imbalance resulted in an increase in worm health and lifespan [43].

## 6. Omega-6 PUFAs Promote Ferroptosis

**Figure 2:**
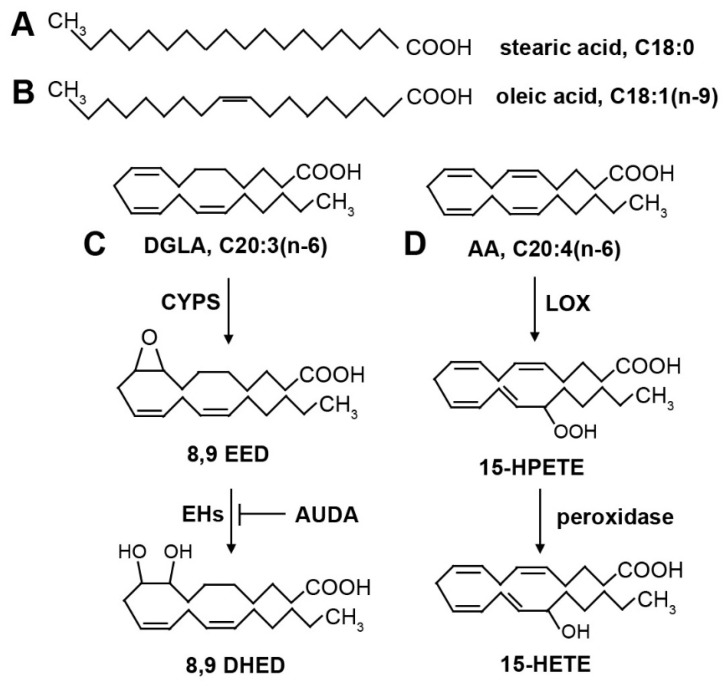
Structures of common fatty acids and oxygenated derivatives. (**A**) Stearic acid (18:0) is a saturated fatty acid. (**B**) Oleic acid (18:1n-9) is a monounsaturated fatty acid. The single double bond is in the cis position, creating a kink in the fatty acid that prevents tight packing of fatty acids and contributes to membrane fluidity. (**C**) Dihommo-γ linolenic acid (DGLA, 20:3n-6) is a polyunsaturated fatty acid. Oxygenated derivatives are produced by cytochrome P450 (CYP) enzymes, forming an epoxide. The double bond that is converted to an epoxide depends on the position-specific isoform of CYP enzymes. The epoxides can be converted into diols by epoxide hydrolase (EH) enzymes. The EH enzymes are inhibited by AUDA. (**D**) Arachidonic acid (AA, 20:4n-6) is a polyunsaturated fatty acid. Shown are examples of oxygenated derivatives produced by lipoxygenase (LOX) enzymes and peroxidase activity. The location of the hydroperoxide is dependent on the position-specific isoform of LOX. The hydroperoxide can be further reduced by peroxidase activity, leading to a bioactive hydroxyl derivative.

PUFAs are the most susceptible lipids to peroxidation during ferroptosis [5,24]. The most abundant cellular PUFAs are members of two families, the omega-6 PUFAs and the omega-3 PUFAs, named for the position of the most terminal double bond on the acyl chain (Figure 2). Both families of PUFAs are essential, playing important roles in the development of the nervous system and cognition, skin barrier function, the immune system, reproduction and other essential physiological functions [46,47]. Typically, high levels of dietary omega-6 fats are associated with inflammation and associated diseases, whereas dietary omega-3 fats are considered to be anti-inflammatory, invoking the suggestion that increased intake of omega-3 PUFAs will lead to a beneficial decrease in the ratio of omega-6 to omega-3 PUFAs in membranes [48]. For example, patients with colon cancer found that those with a higher intake of marine omega-3 PUFAs were associated with longer disease-free survival [49]. However, both omega-3 and omega-6 PUFAs contain multiple double bonds that are susceptible to lipid peroxidation. In an assay of peroxide-induced oxidative stress leading to organismal death in *C. elegans*, dietary supplementation with both omega-6 and omega-3 PUFAs accelerated cellular damage and death [50]. 

Using powerful mass spectrometry methods, Kagan et al. identified oxidized forms of two omega-6 PUFAs, arachidonic acid (AA, 20:4n-6) and adrenic acid (22:4n-6), associated with the endoplasmic reticulum phospholipids in cells undergoing ferroptosis [43]. In acidic cancer cells, excess PUFAs that are not incorporated into lipid droplets are peroxidized and contribute to ferroptosis [51]. Most studies in mammals focus on AA’s role in promoting ferroptosis. In gastric cancer cell lines, PUFA synthesis genes become silenced by DNA methylation, and these cells are resistant to RSL1-induced ferroptosis. Supplementation with AA restored sensitivity to ferroptosis [52]. In C8+ T cell-mediated tumor killing, AA interacts with T cell-generated interferon, contributing to the ferroptotic death of tumor cells [53]. In mice, acetaminophen-induced acute liver failure and cell death is caused by ferroptosis, and was found to be associated with the oxidation of omega-6 PUFAs, particularly AA, and prevented by treatment with the ferroptosis-specific inhibitor Fer-1 [54]. Thus, in mammalian cells, the peroxidation of omega-6 PUFAs, especially AA, is strongly associated with ferroptosis. 

## 7. Germ Cell Surveillance: A Physiological Role for Ferroptosis in *C. elegans*

**Figure 3:**
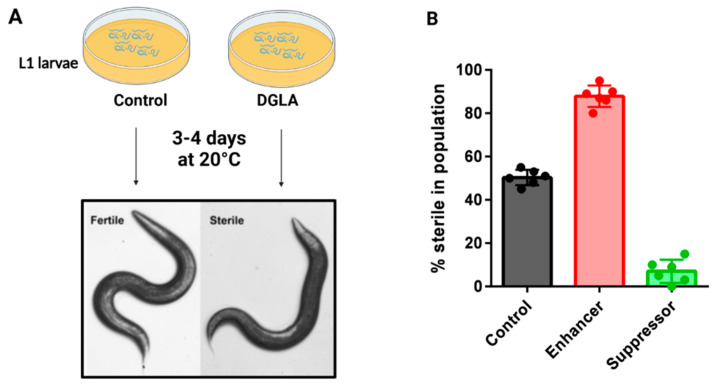
Dietary DGLA causes ferroptosis of germ cells and sterility in *C. elegans*. (**A**) Schematic of the *C. elegans* fatty acid supplementation assay. Synchronized L1 larvae are plated onto agar plates containing DGLA and dietary *E. coli*, and incubated at 20 degrees until they reach adulthood, when they are scored as fertile or sterile. Sterile worms lack gametes due to ferroptosis of germ cells during development. (**B**) Mutant strains that are more sensitive to DGLA are known as enhancers, while mutant strains that are less sensitive to DGLA are known as suppressors. Often, enhancer strains contain mutations in protective genes, such as genes encoding GPX enzymes or genes required for MUFA production. Suppressor genes include genes needed to produce membrane PUFAs, or mutants that confer increased stress responses.

The Watts lab demonstrated that dietary supplementation of the omega-6 PUFA dihomo-γ-linolenic acid (DGLA, 20:3n-6) induces sterility in young *C. elegans* due to the death of germ cells, oocytes and sperm, while somatic cells remained unaffected [55]. When testing the effect of other omega-6 PUFAs, only DGLA and high concentrations of AA led to the sterility phenotype, not other dietary PUFAs such as the omega-3 PUFA eicosapentaenoic acid (EPA, 20:5n-3) [50,55]. Thus, this germ cell death is distinct from the whole-body peroxide stress mentioned above that is promoted by both omega-3 and omega-6 PUFAs. Mutations in genes encoding components of PUFA synthesis pathways, aging and stress resistance modulated the degree of germ cell death [55,56](Figure 3). Specifically, mutant strains with increased endogenous DGLA synthesis and strains with mutations in genes required for lipid homeostasis, such as SREBP and certain nuclear hormone receptors, were most susceptible to DGLA, acting as enhancers (Figure 3B), while strains with blocked PUFA pathways and increased stress resistance activity, such as the long-living *daf-2* mutants, were resistant to DGLA, acting as suppressors (Figure 3B) [55,56]. 

To test whether ferroptosis contributes to germ cell death due to dietary DGLA, worms were treated with the omega-6 PUFA and the ferroptosis-specific radical-trapping antioxidant inhibitor ferristatin-1 (Fer-1) and it was found that both germ cell death and sterility were reduced when compared to worms treated with DGLA alone [57]. Similarly, the antioxidant vitamin E also protected against DGLA-induced cell death. A *gpx-1* mutant strain showed higher susceptibility to DGLA than WT. Cellular iron was manipulated in several ways. First, the *ftn-1* mutant strain lacking the iron storage protein ferritin was shown to be more susceptible to dietary DGLA, while treatment with the iron chelator 2,2′-bipyridine prevented DGLA-induced cell death, demonstrating a role for cellular iron in the cell death process [57]. Taken together, these results demonstrated that dietary DGLA induces ferroptosis in *C. elegans* germ cells, creating a powerful physiological model of ferroptosis that can be studied in a genetically tractable system. We propose that ferroptosis acts as a surveillance mechanism to deplete germ cells when too much oxidative damage is present, therefore ensuring that only undamaged germ cells survive and produce embryos.

## 8. Fatty Acid Composition of Ether Lipids Influences Ferroptosis 

While many types of phospholipids contain PUFAs that can undergo peroxidation during ferroptosis, the phosphatidylethanolamine (PE) lipids are most often implicated [39,43], perhaps because this phospholipid class often contains PUFAs and is confined to the inner leaflet of the plasma membrane. Some PE species contain ether linkages, rather than the usual ester linkages, at the *sn-1* position of the PE phospholipid, known as ether lipids. Ether PLs are understudied, but are proposed to play roles in cellular signaling and membrane structure, and may act as endogenous antioxidants [58]. Dysregulation of ether PLs can lead to diverse human diseases including neurodegenerative diseases and cancer [59]. 

In the *C. elegans* germ cell ferroptosis model, ether lipid-deficient mutant strains are sensitive to DGLA, suggesting a protective role in ferroptosis for ether lipids [57], consistent with a role for ether lipids as endogenous antioxidants. In contrast, several mammalian studies showed that ether lipids drive ferroptosis [60,61]. These studies demonstrated that the depletion of genes required for the synthesis of ether lipids results in decreased levels of PUFA-containing ether PLs and reduced ferroptosis. Supplementation of PUFA-ether PLs in cells deficient in ether PLs resensitized cells to ferroptosis. However, MUFA-containing ether lipids did not induce ferroptosis. The discrepancies between the *C. elegans* and mammalian studies showing opposite roles for ether lipids in the modulation of ferroptosis might be explained by the fatty acid composition of ether phospholipids. While most ether lipids contain a saturated fatty acid connected by an ether bond in the *sn-1* position, mammalian ether lipids most often contain a PUFA on *sn-2*, and *C. elegans* ether lipids most often contain a MUFA in this position [50,62]. Thus, the homeostatic regulation of fatty acyl composition is an important regulator of lipid peroxidation potential and ferroptosis in cells, perhaps more so than the particular class of phospholipid.

## 9. Monounsaturated Fatty Acids Protect Membranes from Ferroptosis

Given ferroptosis’s reliance on lipid peroxidation, the composition of available lipids in a system can play a major role in determining ferroptotic sensitivity. Oleic Acid (OA), a MUFA (Figure 2B), has previously been shown to inhibit ferroptosis in several cell lines, including HT-1080 [24]. MUFAs have since been shown to inhibit ferroptosis by limiting the incorporation of PUFAs into the cellular membrane, and thus limiting the accumulation of lipid ROS formed in an ACSL3-dependent manner [63]. 

These findings have been mirrored in *C. elegans*, whose fatty acid composition is well documented [55,64,65]. MUFAs have previously been shown to promote an increased lifespan in *C. elegans* [66]. The role of monounsaturated fatty acids (MUFAs) in protecting against ferroptosis has been further characterized across several studies. *C. elegans* grown on bacteria supplemented with both the ferroptotic inducer DGLA and the MUFA OA were protected from ferroptosis [57]. Furthermore, the *C. elegans fat-2* mutants, which generate high levels of OA [65], were significantly resistant to even high concentrations of supplemented DGLA [57]. Interestingly, the dependence on ether lipids for ferroptosis resistance was strongly dependent on endogenous MUFA and PUFA synthesis, demonstrating that it was not the presence or absence of ether lipids per se, but rather that ether lipid deficiency in *C. elegans* disrupted membrane homeostasis and led to decreased ratios of MUFA compared to saturated fats and PUFAs in cellular membranes. By restoring membrane MUFAS, ferroptotic cell death was reduced, as well as the levels of the lipid peroxidation product MDA [50]. Thus, in both mammals and *C. elegans,* MUFAs have the potential to therapeutically prevent or limit ferroptosis. 

## 10. Lipid Remodeling Enzymes Influence Membrane Composition and Ferroptosis

Strong evidence indicates that the presence of PUFAs in phospholipids is required for their role in promoting ferroptosis. This is based on the requirement of lipid remodeling enzymes that catalyze the insertion of fatty acids into membrane phospholipids [67,68]. The cleavage and reinsertion of fatty acids into membrane phospholipids occurs continuously in cells in a process known as the Lands cycle [69]. This process promotes the removal of peroxidized fatty acyl groups and the insertion of new fatty acids in their place. Members of the phospholipase A2 family are especially relevant in ferroptosis because they specifically remove fatty acids at the *sn-2* position, the preferred location of PUFAs in phospholipids [70]. In p53-driven ferroptosis, iPLA2β removes peroxidized lipids to suppress cell death, while the depletion of endogenous iPLA2β sensitizes tumor cells to ROS-induced ferroptosis [71]. To be incorporated into cellular membranes, long-chain fatty acids need to be converted to their respective acyl-coenzyme A (acyl-CoA) forms, which is usually initiated by acyl-CoA synthetases (ACSL), and incorporation occurs via acyl transferase enzymes (LCAT). Indeed, impairing ACSL4 and LCAT3 activity suppresses ferroptosis in multiple systems [67,72,73]. On the other hand, knockdown of ACSL3 enhances ferroptosis [63]. The differences in ferroptosis modulation depend on the substrate specificity of the acyl-CoA synthetase enzymes; ACSL4 prefers long-chain PUFA substrates, while ACSL3 prefers MUFAs [39].

However, the requirement for ACSL4 in ferroptosis is not universal. The Dixon group compared multiple loss-of-function genetic screens using a range of cell types and a range of ferroptosis induction mechanisms from various studies. They identified only a handful of genes that were required for ferroptosis across a range of different screens. Intriguingly, disrupting ACSL4 resulted in a greater suppression when ferroptosis was triggered by direct GPX4-inhibition compared to other forms of ferroptosis induction, such as by cystine deprivation or by the iron oxidizing agent FINO_2_ [74]. This indicates a context-specific role for ACSL4 and other ferroptosis regulatory genes, making it difficult to establish a unifying key effector of ferroptosis, other than the convergence of lipid peroxidation on the plasma membrane as the ultimate damage resulting in cell death. 

## 11. The Ability of Specific PUFAs to Induce Ferroptosis May Depend on Enzymatic Conversion by Lipoxygenases or CYPs

A key aspect of ferroptosis, as the name implies, is the dependance on iron [1]. As mentioned above, during non-enzymatic lipid peroxidation, iron contributes to the Fenton reaction that generates lipid radicals that can initiate or help propagate the peroxidation cascade in membranes. On the other hand, enzymes involved in specific lipid peroxidation reactions, such as lipoxygenases and cytochrome P450 oxidoreductase activities, often rely on ferrous iron (Fe^2+^) as a cofactor. The role of some of these iron-requiring PUFA modification enzymes has been studied in the context of ferroptosis.

Lipoxygenases are enzymes that use iron and oxygen to catalyze the stereo-specific dioxygenation of PUFAs at specific locations in the acyl chain, producing hydroxy and peroxy PUFA derivatives that serve as lipid mediators in many biological processes [75,76]. The most-studied LOXs in mammals use AA as a substrate to generate bioactive lipid mediators such as 15-hydroxyeicosatetraenoic acid (15-HETE) and other oxygenated AA derivatives, hence these enzymes are often called ALOX (Figure 2D). ALOXs have been examined in ferroptosis, and appear to have context-specific roles. Silenced ALOX genes in human cells with ferroptosis inducers showed differing results: cells with decreased glutathione demonstrated resistance to ferroptosis, while cells with GPX4 directly inhibited did not demonstrate a change in resistance to ferroptosis [24]. In cancer cells, 12/15 Lox inhibitors of were shown to lower the level of apparent ferroptotic cell death, while overexpression or inhibition of a specific ALOX gene, ALOX15, appeared to increase and decrease the levels of cell death, respectively [77]. The applicability of manipulating ALOX enzymes in ferroptosis has been questioned because the ALOX enzymes may not be highly expressed in cancer cell lines that can undergo ferroptosis [78], and it has been suggested that lipoxygenase inhibitors can also confer radical trapping activity [79]. Interestingly, in conjunction with the PE binding protein 1 (PEBP1), ALOX15 generates 15-hydroperoxy-eicosatetraenoyl-PE (15-HpETE-PE), which confers pro-ferroptotic activity [43]. This enzyme complex can also be inhibited by the radical-trapping antioxidant ferrostatin-1 [80]. Thus, the roles of ALOX in ferroptosis have not been fully resolved and may be context-specific.

Cytochrome P450s are a large group of heme-containing oxidation enzymes [81,82]. In conjunction with cytochrome P450 reductase, CYPs are known to convert various PUFAs into a wide range of oxygenated products, including lipid mediators such as hydroxides and epoxides (Figure 2C) [83,84]. CYPs have been shown to result in the formation of ROS through substrate binding and redox reactions involving the heme group (containing a ferric iron) inside the CYP enzyme [85].

Cytochrome P450 reductases (POR) are oxidoreductases that contribute to a variety of cellular mechanisms such as steroid metabolism and the breakdown of xenobiotics [86]. PORs have been implicated to play a role in lipid peroxidation and ferroptosis. First, POR has been identified in CRISPR screens as enabling ferroptosis [7]. POR and the NADH-cytochrome b5 reductase (CYB5R1) were shown to be required for ferroptosis in a way that does not depend on interactions with CYPs, instead by producing hydrogen peroxide to initiate an iron-dependent Fenton reaction that induces lipid peroxidation, leading to membrane rupture in liposomes during ferroptosis [87]. Additionally, ferroptotic inhibitors such as ferrostatin-1 decreased the level of lipid peroxidation produced by both POR and CYP5R1, implicating the role of oxidoreductases in ferroptosis [87].

## 12. DGLA-Induced Ferroptosis in *C. elegans* Mediated by CYP Activity 

*C. elegans* contains many CYP homologs. One of these, CYP-33E2, has the ability to convert DGLA into epoxide products [88,89](Figure 2C). To explore the bioactivity of DGLA-derived lipid mediators, epoxides were injected directly into the gonads of *C. elegans* and shown to lead to plasma membrane destruction in the gonad, similar to that seen with DGLA injection [89]. While non-enzymatic lipid peroxide propagation is almost certainly occurring in the DGLA-induced ferroptosis model, the enzymatic conversion of DGLA to an epoxide could be a first step in inducing ferroptosis. An enzymatic induction step is a plausible explanation for the specificity of dietary PUFA induction of cell death, in particular why ferroptosis is not triggered by dietary ingestion of more highly unsaturated PUFAs such as eicosapentaenoic acid (EPA, 20:5).

In addition to DGLA inducing ferroptosis of germ cells in young *C. elegans* worms, neurons are also affected by dietary DGLA, but not EPA [90]. Dopaminergic neurons, and, to a lesser extent, glutaminergic neurons, exhibited neurodegeneration in middle-aged worms after DGLA supplementation by a mechanism consistent with ferroptosis. A dihydroxy metabolite of DGLA, produced in two steps by conversion of DGLA to an epoxide, then to a diol by an epoxide hydrolase activity (Figure 2C), was also capable of inducing ferroptotic cell death in dopaminergic neurons [90], demonstrating that a lipid mediator derived from DGLA is initiating ferroptosis in specific cell types. This demonstrates the power of *C. elegans* to tease out the roles of PUFAs and PUFA-derived lipid mediators in ferroptosis in a live animal system. 

## 13. Conclusions

PUFAs play central roles in ferroptosis due to their propensity to form peroxyl radicals that propagate by chain reaction throughout a membrane, leading to irreparable membrane damage and cell death. While membrane PUFAs are protected from peroxidation by several mechanisms, the misregulation or depletion of these protective enzymes and molecules lead to excess peroxidation and ferroptotic damage. Iron contributes to the promotion of lipid peroxidation in both the autoxidation pathway and by acting as a cofactor for enzymatic peroxidation. Studies in *C. elegans* and other cell models suggest that ferroptosis can be triggered by enzymatic conversions of PUFAs, although membrane damage is likely propagated by autoxidation. 

## Data Availability

Data sharing not applicable.
